# Determining the Impact of Hogget Breeding Performance on Profitability under a Fixed Feed Supply Scenario in New Zealand

**DOI:** 10.3390/ani11051303

**Published:** 2021-04-30

**Authors:** Lydia J. Farrell, Paul R. Kenyon, Peter R. Tozer, Stephen T. Morris

**Affiliations:** 1 Teagasc Animal & Grassland Research and Innovation Centre, Mellows Campus, Athenry H65 R7, Ireland; 2 School of Agriculture and Environment, Massey University, Private Bag 11 222, Palmerston North 4442, New Zealand; P.R.Kenyon@massey.ac.nz (P.R.K.); P.Tozer@massey.ac.nz (P.R.T.); S.T.Morris@massey.ac.nz (S.T.M.)

**Keywords:** flock dynamics, system dynamics, profit, bio-economic, modelling, sheep, ewe lambs, east coast, hill country

## Abstract

**Simple Summary:**

In New Zealand, hoggets (female lambs aged 4 to 16 months) can be bred at 8 to 9 months of age to produce a lamb. Breeding hoggets may improve farm production and profit but their levels of production are highly variable. This study modelled ewe flocks with combinations of hogget and mature ewe production levels to investigate changes in production and profit with hogget breeding. Firstly, breeding hoggets was profitable even when hogget production levels were as low as 0.26 lambs weaned per hogget. Secondly, improving mature ewe production levels was more beneficial for profit than larger improvements in hogget production levels. Thirdly, the highest profit was achieved when a flock had both very high mature ewe and hogget production levels. Fourthly, there was a mature ewe production level with which profit was the same for a flock without hogget breeding compared with a flock achieving New Zealand industry average hogget and mature ewe production levels. Overall, the relative profit levels achieved by the modelled flocks suggest more farmers should consider breeding hoggets but improving mature ewe production levels should be prioritised.

**Abstract:**

Hoggets (ewe lambs aged 4 to 16 months) can be bred from approximately 8 months of age for potentially increased flock production and profit, however most New Zealand hoggets are not presented for breeding and their reproductive success is highly variable. Bio-economic modelling was used to analyse flock productivity and profit in four sets of scenarios for ewe flocks with varying mature ewe (FWR) and hogget (HWR) weaning rate combinations. Firstly, hogget breeding was identified to become profitable when break-even HWRs of 26% and 28% were achieved for flocks with FWRs of 135% and 150%, respectively. Secondly, relatively smaller improvements in FWR were identified to increase profit to the same level as larger improvements in HWR. Thirdly, a high performing flock with FWR and HWR both ≥ the 90th percentile currently achieved commercially, was the most profitable flock modelled. Fourthly, a FWR was identified with which a farmer not wishing to breed hoggets could have the same profit as a farmer with a flock achieving current industry average FWR and HWR. Overall, the relative profit levels achieved by the modelled flocks suggest that more farmers should consider breeding their hoggets, though improvements in FWRs should be prioritised.

## 1. Introduction

Hoggets (ewe lambs aged 4 to 16 months) can be successfully bred from approximately 8 months of age and in 2019 33% of hoggets in New Zealand were presented for breeding [1]. Production advantages of breeding hoggets include: (1) increased utilisation of spring pasture, (2) higher numbers of lambs weaned and increased income through more lamb sales, (3) a tool to increase the rate of genetic gain, and (4) reduced intensity of emissions of greenhouse gas per kg of product [2,3,4,5,6,7,8,9,10,11,12]. However, various producer concerns have resulted in most New Zealand sheep farmers choosing not to breed their hoggets. Cited concerns include: (1) poor and varied hogget reproductive performance; (2) potential for lighter live weights and lower survival to weaning of lambs born to hoggets; (3) potential for negative impacts for future productivity and live weight of bred hoggets; (4) greater total sheep feed demand likely requiring changes in numbers of other stock classes; and (5) uncertainty regarding economic outcomes [3,7,8,10,11,12,13,14].

Previous bio-economic modelling of Australian ewe flocks identified hogget breeding to increase farm profit by a lesser amount than improvements in mature ewe reproductive success and optimisation of lamb sale dates [15,16]. Farrell et al. [17] identified a HWR (rate of lambs weaned per hogget presented for breeding) as low as 60% to be more profitable than no hogget breeding, suggesting there may be a ‘break-even’ HWR below 60% where hogget breeding is no longer profitable. They also suggested improvements in FWR (rate of lambs weaned per mature ewe presented for breeding) could provide more economic benefits than achieving a high HWR for many New Zealand farmers [17]. However, there may be a relatively high HWR with which a flock achieving industry average FWR and breeding hoggets could match the profitability of a flock with a high FWR not breeding hoggets. Without this knowledge farmers cannot make informed decisions regarding hogget breeding.

The objective of this analysis was to model several sets of scenarios with varying hogget (HWR) and mature ewe weaning rates (FWR) to estimate changes in sheep enterprise profitability. Specifically to (1) identify the ‘break-even’ HWR where breeding hoggets became profitable for a given FWR; (2) identify the HWR with which hogget breeding for a flock with the current industry average FWR increases profit more than an increase in FWR from the average FWR to the 90th percentile FWR; (3) estimate the potential profitability of a high performing flock achieving both FWR and HWR in the top 10th percentile; and (4) identify the FWR with which the profit of a flock without hogget breeding matches that of a flock with industry average FWR and HWR. Model output for these scenarios will provide important information to famers on the profitability of hogget breeding to aid their decision making regarding this management option.

## 2. Methodology

The New Zealand farm system analysed was an East Coast North Island hill country farm. Assumptions around farm and ewe flock characteristics were informed by industry average values from survey data for this farm system in 2019/20 [18]. This farm of 530 ha had both sheep and beef production enterprises, and sheep account for 60% of total annual farm feed supply and the remainder is consumed by the beef herd [18]. The self-replacing ewe flock was 2255 Romney ewes lambing in spring each year and extensively grazing pasture year-round. This research focused on the sheep enterprise, which produces coarse wool (with a fibre diameter greater than 30 µm) and lambs for meat. The proportion of total farm feed supply consumed by sheep informed assumptions of operating expenses and farm area accounted for by the ewe flock and sheep enterprise. Total annual sheep feed demand was fixed to 60% of total feed supply in all modelled scenarios so the beef enterprise was assumed not to be affected by any changes modelled in the sheep enterprise.

This analysis used a bio-economic system-dynamics model which was run in STELLA 1.7.1 [19] and this model was previously used to investigate changes in the production and profitability in New Zealand sheep enterprises with system changes [20,21]. Details on flock dynamics, wool production, and estimation of sheep feed demand have been published by Farrell et al. [17]. Details on lamb production, lamb sales, sheep enterprise economics, and lamb weaning rates are provided in the following subsections.

### 2.1. Lamb Production and Sale Policies

Mature ewes began lambing on 1 September and hoggets began lambing a month later [8,22]. Ewes weighed 65 kg and hoggets weighed 80% of ewe weight at one year of age [23,24,25]. Foetal and lamb losses between pregnancy diagnosis and weaning were assumed to be 15% for lambs from mature ewes and 25% for lambs from hoggets [23,24,25,26,27]. Lamb weights at birth were assumed to be 5.5 kg and 4.5 kg for single-born and multiple-born lambs born to mature ewes, respectively [28,29]. Birth weights for those born to hoggets were assumed to be 4.5 kg for single-born lambs and 3.9 kg for multiple-born lambs [23,24,28,29]. Lambs born to mature ewes were assumed to be weaned at 12 weeks old with weights of 30 kg for single-born lambs and 28 kg for multiple-born lambs [30,31]. Lambs from hoggets were 10 weeks old at weaning and weighed 23 kg for single-born lambs and 17.4 kg for multiple-born lambs [32].

Lambs born to mature ewes were sold in three separate groups, of which two groups of lambs were sold prime (sold direct to slaughter), then the third group was sold store (for a different farmer to grow to slaughter weight). The prime lambs had a carcass weight of 18 kg on average at sale [18] with a carcass dressing out rate of 41% [33]. Sales of prime lambs born to mature ewes occurred in early February and mid-February based on the estimated average post-weaning growth rate of 100 g/day [22,34,35]. The third group of lambs born to mature ewes was sold store in mid-February weighing 30 kg, lighter than prime lambs would typically be sold [36]. All lambs born to hoggets were sold store mid-December, which was less than two weeks after weaning, weighing 24 kg after growing 100 g/day, on average [34,35].

### 2.2. Income, Expenses, and Profit

All prices, income, expenses, and profit values were in New Zealand dollars (NZD). Cash operating surplus (COS) of the sheep enterprise was used as a profit indicator in this analysis, estimated per ha according to income (from sales of sheep and wool) and operating expenses relating to the sheep enterprise. Beef enterprise profit was assumed not to change between modelled scenarios as sheep feed demand was fixed at 60% of total farm feed supply annually. Sheep sale prices from 2019/20 were used (Table 1) in estimations of income.

Expenses related to animal health were NZD 6.00/SU (stock unit) and the remaining operating expenses included in this analysis were NZD 48.19/SU [18]. Operating expenses included variable costs and the sheep enterprise share of fixed costs (comprising of expenses for ACC (Accident Compensation Corporation levy), vehicles, repairs and maintenance, insurance, and administration) and excluded tax, drawings, depreciation, rent, and interest [37]. Ewes, rams, and replacement lambs were included in sheep stock units for expense estimations. Each stock unit was approximately 5500 MJ ME (metabolisable energy) annually [38], and was adjusted for mature ewes and hoggets based on their liveweight and weaning rate [39]. Shearing expenses were NZD 10.98 per ewe and NZD 3.71 per lamb born to mature ewes (lambs born to hoggets were sold before shearing in January) [40].

Breeding expenses, such as ram purchases, were included in operating expenses for mature ewes. Ram purchase costs for hoggets were estimated separately. Ram prices were assumed to be NZD 800 each with each ram used for three years (NZD 267 each per year) [40]. Rams were purchased at a ratio of 75 hoggets per teaser ram (vasectomised ram for pre-breeding) and 50 hoggets per breeding ram [11,41,42]. The direct expenses relating to hogget breeding including in this analysis were increased stock unit per hogget (with increased weaning rate) and ram purchases.

### 2.3. Flock and Hogget Weaning Rates

The first set of modelled scenarios identified the break-even HWRs where hogget breeding became profitable for a given FWR. The flock weaning rate (FWR) was modelled at two levels: FWR = 135%, the average 2019/20 rate for the New Zealand East Coast North Island hill country farm system under analysis [18], and then at FWR = 150% which was the 90th percentile for 2019/20 weaning rates for this farm system [43]. For analysis of the profitability of hogget breeding, HWR was first set at 0% for each FWR to establish the base COS value for each FWR without hogget breeding. The flock was then modelled with each FWR and increasing HWR until the COS with hogget breeding was equal to COS without hogget breeding.

The second set of scenarios identified the HWR with which hogget breeding increased profit more than an increase in FWR from the current industry average FWR to the 90th percentile FWR. This analysis further explored previous findings that a flock achieving the average 2017/18 FWR and the relatively high HWR of 100% was less profitable than a flock achieving the 90th percentile FWR and not breeding hoggets [17]. In the current analysis the flock was modelled with the 90th percentile FWR of 150% and no hogget breeding to determine the resultant COS value. The flock was then modelled with FWR = 135% and increasing HWR until the same COS value was obtained. This determined the theoretical HWR (>100%) required to justify a farmer focusing on improvements in hogget reproductive success over that of mature ewes. 

Thirdly, to estimate the profitability of a flock achieving both FWR and HWR in the top 10th percentile, representing a high performing New Zealand flock. The flock in this scenario was modelled with FWR = 150% and the high HWR (>100%) modelled in the second set of analyses. 

The fourth scenario identified the FWR with which a flock not breeding hoggets could match the COS of a flock achieving industry average FWR and HWR. The flock was modelled with the average 2019/20 FWR = 135% [18] and approximate average HWR = 60% [1,44] to identify the resultant COS value. The flock was then modelled with increasing FWR and no hogget breeding until the same COS level was achieved. 

In all modelled scenarios, it was assumed all hoggets on-farm were presented for breeding, thus total hogget numbers were the denominator for HWR.

## 3. Results and Discussion

Model output is shown and discussed in the following subsections for chosen scenarios with various combinations of mature ewe (FWR) and hogget (HWR) weaning rates relevant to farmers considering hogget breeding. Explored scenarios allow for determination of the impacts of hogget breeding on flock size, production, feed demand, and profitability. Output for these scenarios is displayed in figures in the order they were presented in Section 2.3 and discussed in Section 3.4.

### 3.1. Sheep Numbers 

**Figure 1:**
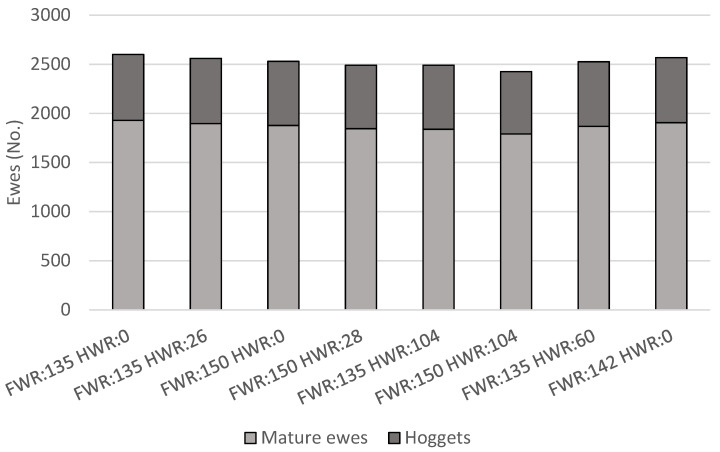
Numbers of mature ewes (2 to 6 years old) and hoggets (1 year old), for scenarios with combinations of varying weaning rates (weaned lambs per ewe that was presented for breeding) of mature ewes (FWR) and hoggets (HWR).

**Figure 2:**
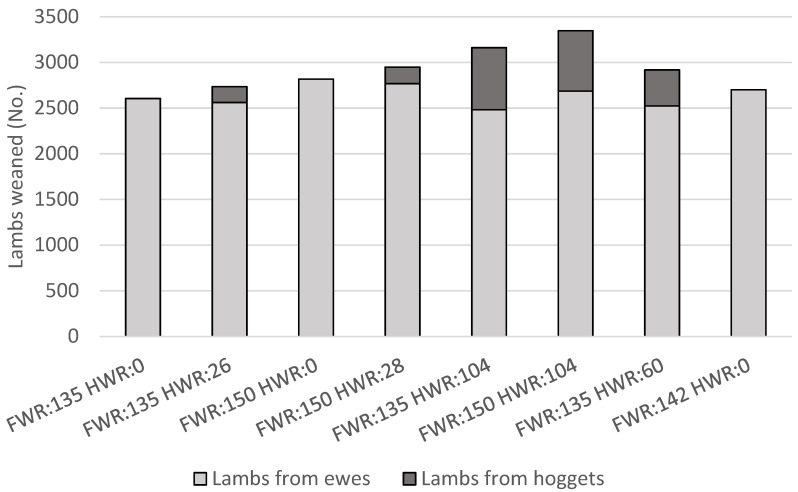
Weaned lambs from mature ewes (2 to 6 years old) and weaned lambs from hoggets (1 year old), for scenarios with combinations of varying weaning rates (lambs weaned per ewe presented for breeding) of mature ewes (FWR) and hoggets (HWR).

The ewe flock was larger, 2600 total sheep (671 hoggets and 1929 mature ewes) with FWR = 135% and HWR = 0% compared to 2530 sheep (653 hoggets and 1877 mature ewes) with FWR = 150% and HWR = 0% (Figure 1). With the FWR = 150% the flock had a higher per ewe feed demand (for gestation and lactation of their lambs), therefore the total flock size was smaller to maintain sheep feed demand at 60% of feed supply. Similarly, for a given FWR there were fewer total sheep in flocks with hogget breeding, compared to not hogget breeding, due to the increased per hogget feed demand necessitating reductions in flock size. The smallest flock size, 2425 sheep (633 hoggets and 1790 mature ewes) occurred with FWR = 150% and HWR = 104% (Figure 1). Ewes in this scenario also weaned the most lambs in total (3347 lambs; Figure 2), therefore, with the higher per ewe and per hogget feed demand (for the gestation and lactation of their lambs) the smaller flock size was expected. 

### 3.2. Lamb Production

The highest total lamb production, 3347 weaned lambs, was achieved with FWR = 150% and HWR = 104% (Figure 2). As HWR increased so did the proportion of total lambs which were weaned from hoggets. The highest proportion of total weaned lambs in the flock which were born to hoggets was 21%, occurring with the lowest FWR (FWR = 135%) and highest HWR (HWR = 104%) modelled. This was higher than the average proportion of lambs weaned from hoggets, as a proportion of total lambs weaned, for the farm system under analysis in the 2019/20 production year (6.7%) [18], although this industry average included farms that did not breed hoggets. 

For a given FWR, numbers of total weaned lambs increased with hogget breeding, even with relatively low HWRs and reductions in flock size. For example, with FWR = 150% and HWR = 0%, 2817 total lambs weaned, fewer than with FWR = 150% and HWR = 28% where 2948 total lambs were weaned. With FWR = 150% and HWR = 28% there were 181 lambs weaned from hoggets, but 50 fewer lambs were weaned from mature ewes compared with FWR = 150% and HWR = 0%, therefore 131 more lambs were weaned in total. The total per lamb feed demand for lambs born to hoggets was lower than that of lambs from mature ewes, due to lower birth weights resulting in lower demand for gestation, and lower weaning weight and weaning at a younger age resulting in lower demand for lactation. Further, in these scenarios lambs born to hoggets were sold store soon after weaning with less demand for post-weaning maintenance and growth compared with lambs born to mature ewes.

### 3.3. Sheep Feed Demand

**Figure 3:**
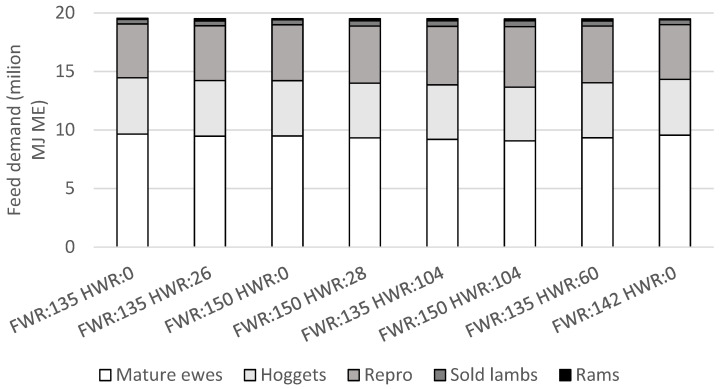
Annual feed demand (metabolisable energy) of mature ewes (2 to 6 years old), hoggets (1 year old ewes), reproduction (gestation and lactation of all lambs), sold lambs prior to leaving the farm, and rams, for scenarios with combinations of varying weaning rates (lambs weaned per ewe which was presented for breeding) of mature ewes (FWR) and hoggets (HWR).

Total sheep feed demand was fixed at 60% of feed supply annually in all scenarios, the average proportion of feed consumed by sheep for this farm type [18], at approximately 19.5 million MJ ME (Figure 3). The proportion of total sheep feed demand accounted for by the maintenance demand of the mature ewe flock decreased as numbers of weaned lambs increased (Figure 2 and Figure 3), as occurred with hogget breeding, due to the smaller flock size and increased feed demand for lamb production (for gestation and lactation). When the maintenance feed demand of mature ewes was combined with the feed demand for maintenance and growth of replacement hoggets, their combined feed demand accounted for approximately 72% of total annual sheep feed demand (Figure 3). Feed demand for lamb production included gestation, lactation, and the post-weaning growth and maintenance of sold lambs until they left the farm. Feed demand for lamb production averaged 27% of total annual sheep feed demand, ranging from 26% to 29%, and was lower without hogget breeding. Emissions of greenhouse gases on a per kg of product basis can be reduced in animal production systems as the proportion of feed used for production increases, reducing the proportion of feed used for animal maintenance [9]. This has been identified to occur with higher weaning rates and younger ewe age at first lambing [9,45]. Therefore, hogget breeding and achieving greater hogget reproductive success as observed in some of the modelled scenarios, has the potential to increase the efficiency of, and decrease the emissions intensity of, lamb production systems.

Feed demand for rams was approximately doubled with hogget breeding due to requirements for additional rams (Figure 3). When rams were only required for breeding with the mature ewe flock their annual feed demand averaged 85,000 MJ ME (0.4% of total annual sheep feed demand), this increased to an average of 180,000 MJ ME (0.9% of total annual sheep feed demand) when additional rams were on-farm for teasing and breeding of hoggets.

### 3.4. Economics

**Figure 4:**
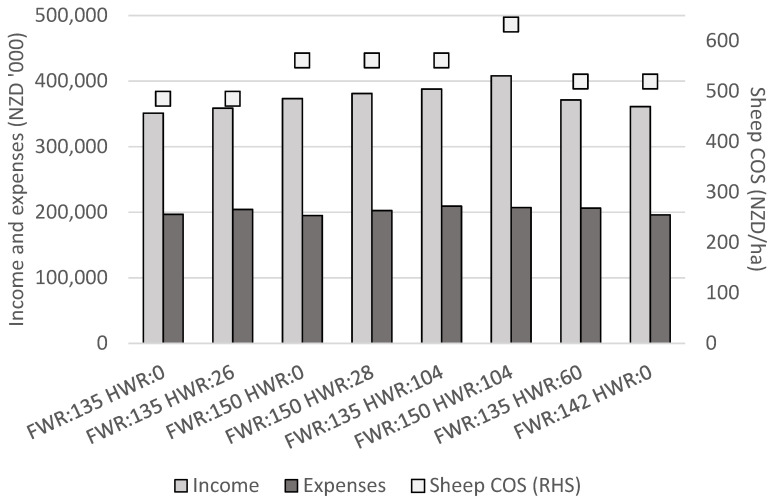
Income from sales of sheep and wool, expenses, and cash operating surplus (COS), for scenarios with combinations of varying weaning rates (lambs weaned per ewe which was presented for breeding) of mature ewes (FWR) and hoggets (HWR). RHS = right hand side axis.

The sheep enterprise COS achieved in the modelled scenarios ranged from NZD 485/ha to NZD 632/ha (Figure 4). The average EBITR for this type of farm system in the 2019/20 production year was between NZD 500/ha and NZD 600/ha [43]. Therefore, the COS levels estimated in this analysis were sufficiently similar that it could be assumed the scenarios were representative of the farm system in New Zealand in the 2019/20 production year.

#### 3.4.1. Break Even Hogget Weaning Rates

The purpose of the first set of scenarios in this analysis was to determine the ‘break-even’ HWRs for sheep enterprise COS to be the same value with and without hogget breeding, for a given FWR and the same total annual sheep feed demand. This analysis compared a flock achieving FWR = 135% and HWR = 0% with a flock achieving FWR = 135% and HWR = 26%, then another comparison was made between a flock achieving FWR = 150% and HWR = 0% with a flock achieving FWR = 150% and HWR = 28% (Figure 4). 

Hogget breeding displaced mature ewes from the flock in order to maintain total annual sheep feed demand, for the flock with FWR = 150% a more productive ewe was displaced, therefore, the ‘break-even’ HWR was higher (at 28%) than for the scenarios with FWR = 135% (break-even HWR = 26%). COS was NZD 485/ha in the base scenario with FWR = 135% and HWR = 0%, and NZD 561/ha in the scenario with FWR = 150% and HWR = 0%. COS was also NZD 485/ha with FWR = 135% and HWR = 26% and COS was NZD 561/ha with FWR = 150% and HWR = 28%. This suggested the additional income from sales of lambs born to hoggets with FWR = 135% and HWR = 26% was equal to the additional expenses incurred, including the reduction in sales of lambs from mature ewes and, thus, that with HWR ≥ 26% hogget breeding would be more profitable. The additional expenses for hogget breeding included in this analysis were purchases of additional rams, increased per head stock unit for hoggets, increased total hogget deaths, and the opportunity cost of farming fewer mature ewes. Differences in expenses between scenarios were relatively small, as illustrated in Figure 4 where expenses were either slightly under or over NZD 200,000 without or with hogget breeding, respectively. Changes in COS were therefore largely the result of changes in income, and 90% of total annual sheep enterprise income was accounted for by income from sheep sales (the remainder being wool sales). With FWR = 135% and HWR = 26% there were 181 lambs weaned from hoggets, generating NZD 14,206 of additional income (Figure 2 and Figure 4). Reductions in ewe numbers (compared with FWR = 135% and HWR = 0%) reduced income from sales of lambs from mature ewes by NZD 5858 (Figure 4). Purchases of rams for hogget breeding (excluding expenses for rams bred with mature ewes) incurred expenses of NZD 5500. Therefore, the break-even HWR of 26% was largely explained by changes in total income from lamb sales and expenses incurred by purchases of additional rams, with small changes in wool income and total operating expenses accounting for the remainder of changes in income and expenses due to hogget breeding.

The identified break-even HWRs of 26% and 28% suggest hogget breeding should be profitable on New Zealand farms with these relatively low HWRs achieved (Figure 4). This suggests more New Zealand farmers should consider breeding their hoggets to increase total numbers of lambs weaned in New Zealand and sheep enterprise profitability. National statistics indicate that only 33% of the total hoggets in New Zealand were presented for breeding in 2019 and these hoggets weaned lambs at a rate of 56% [1]. Responses from New Zealand sheep farmers to a questionnaire in 2002 indicated HWR to average 60%, and 6% of surveyed farmers achieved HWR ≥ 100% [44]. According to the 2002 survey data HWR = 28% was around the lower 10th percentile of weaning rates achieved on-farm [44] and the same survey identified North Island Hill Country farms, the system under study in the current analysis, to be the second-most likely type of sheep farm to breed their hoggets [8]. New Zealand industry data suggest the majority of farmers breeding hoggets achieve HWRs greater than 28% and would likely not consider hogget breeding if such a low HWR were only achieved. Further, although additional expenses incurred by hogget breeding were included in the study, it is acknowledged that there may remain associated indirect costs not accounted for in the current analysis. These could include flow-on effects of the additional labour requirements of hogget breeding and any potential negative impacts on the future reproductive success and longevity of bred hoggets, both of which have been cited by farmers as deterrents from hogget breeding [8]. The ‘break-even’ HWRs where hogget breeding was identified to be more profitable above these HWRs in this analysis may therefore not be acceptable HWRs for farmers and may explain why the majority of New Zealand farmers do not choose to breed their hoggets. However, if these ‘break even’ HWRs were known by farmers, more farmers may choose to breed hoggets to improve farm profitability.

The current analysis assumed all hoggets were presented for breeding, therefore the denominator in HWR included all on-farm hoggets. An equivalent HWR to the 26% or 28% break-even HWRs for flocks modelled with FWR = 135% or FWR = 150%, respectively (Figure 4), in this analysis could potentially be achieved through presentation of only a proportion of on-farm hoggets for breeding, with presented hoggets achieving a similar HWR to the industry average of 60% [1,44]. The farmer survey HWR values were based only on hoggets presented to the ram [44]; however, the national statistics suggest a rate of total lambs weaned from hoggets divided across total on-farm New Zealand hoggets to be 19% in 2019 [1]. As stated above, North Island hill country farms were more likely to present hoggets for breeding [8], therefore the break-even HWRs of 26% and 28% estimated in this analysis may be similar to those achieved on commercial farms of this type presenting just a selection of their hoggets for breeding. If farmers present only a proportion of their hoggets for breeding, such as the heaviest animals, this would require fewer rams and therefore reduced feed and expenses incurred by hogget breeding. Changes in feed and expenses for ram purchases may also change the ‘break-even’ HWRs from those estimated in this analysis. Hoggets could then be managed in two separate flocks from pre-breeding onwards (at 8 to 9 months of age in autumn): one flock of heavier hoggets to be presented for breeding, and another flock of lighter hoggets not to be bred until approximately 19 months of age to lamb as two-year olds, which could grow more slowly and consume less autumn and winter feed. 

#### 3.4.2. Improving Hogget or Mature Ewe Weaning Rates

The second set of scenarios aimed to identify a theoretical HWR (>100%) required to justify a farmer focusing on improvements in hogget reproductive success over that of mature ewes. Scenarios for comparison were a flock with FWR = 150% not breeding hoggets and a flock with FWR = 135% and HWR = 104% (Figure 4). Sheep enterprise COS was NZD 561/ha in the scenario with FWR = 150% and HWR = 0% and also in the scenario with the average industry 2019/20 FWR = 135% and HWR = 104% (Figure 4). The difference in FWRs (15%) was proportionally smaller than the difference in HWRs (104%) to achieve the same COS. The expenses associated with increasing FWR were lower relative to increasing HWR as the required ratio of rams to mature ewes was lower and vasectomized teaser rams were not needed for mature ewes. From a farm management perspective, it would likely be easier to improve FWR as modelled compared with HWR, as HWR is more likely to be low and varied [11]. Further, industry data show mature ewes can achieve well above FWR = 150% [43] while the proportion of farms with hoggets achieving weaning rates ≥ 104% has been less than 6% [44]. These results support the conclusions of [15,16,17] that improvements in FWR should be prioritised when considering hogget breeding as a strategy for increased sheep farm profitability. A flock achieving a high HWR (i.e., >100%) would likely achieve a similarly high FWR (i.e., above the average FWR = 135%) as mature ewe and hogget reproductive performances are genetically linked in a self-replacing flock, therefore this scenario (a flock with FWR = 135% and HWR = 104%) may not represent a realistic combination of FWR and HWR occurring on commercial farms. 

#### 3.4.3. A High Performing Flock Breeding Hoggets

For the third set of scenarios a flock was modelled with high (≥90th percentile) FWR and HWR (HWR = 104%), representing a high performing flock breeding hoggets. The highest overall sheep enterprise COS was achieved in this scenario at NZD 632/ha (Figure 4), this was expected as the highest number of total lambs were weaned (3347 lambs; Figure 2) and COS was driven by lamb sales. In the first set of scenarios (Section 3.4.1) it was identified that hogget breeding became profitable with HWR = 28%, for a flock with FWR = 150%. Therefore, although highly productive (FWR = 150%) mature ewes were displaced from the flock with hogget breeding in this scenario, hoggets were themselves also highly productive (HWR = 104%, well above the break-even level of 28%) and so the greatest number of total lambs weaned and sold was achieved in this scenario and also the highest COS.

#### 3.4.4. Forgoing Hogget Breeding

The fourth set of modelled scenarios aimed to determine the FWR without breeding hoggets which could match the COS of the average flock breeding hoggets (achieving both industry average FWR and HWR). This analysis modelled a flock with FWR = 135% and HWR = 60%, the average weaning rates for farms of the type under study [18,43], compared with a flock achieving FWR = 142% and not breeding hoggets (Figure 4). The modelled flock with FWR = 135% and HWR = 60% had a sheep enterprise COS of NZD 519/ha (Figure 4) and had 14% of total lambs weaned as those weaned from hoggets (Figure 2). The same COS value of NZD 519/ha was obtained with FWR increased to 142% and no hogget breeding. The results suggest the sheep enterprise COS achieved with industry average mature ewe and hogget weaning rates could also be achieved by increasing FWR to 142% from 135% and without breeding hoggets. A FWR of 142% was approximately the 75th percentile of weaning rates achieved by farms of the type under study in 2019/20 [43]. The results of this analysis suggest that farmers who do not currently breed hoggets should first prioritise increasing FWR, i.e. to an above-average level such as the FWR of 142% identified here, before focusing on hogget breeding as a means of improving farm profitability. These results can inform farmers who would prefer to focus on improving FWR rather than breeding their hoggets. The COS achieved in this scenario (NZD 519/ha when FWR = 142% and HWR = 0%) was lower than was achieved in the previous scenario of a flock with FWR = 150% and HWR = 0% (where COS was NZD 561/ha), as was expected due to the flock with higher FWR (150%) weaning and selling more lambs. 

## 4. Conclusions

This research explored several sets of scenarios with varying hogget (HWR) and mature ewe weaning rates (FWR) and the same sheep enterprise profitability, as indicated by Cash Operating Surplus (COS). Firstly, ‘break-even’ HWRs were identified to be 26% or 28% for flocks with FWRs of 135% or 150%, respectively, where hogget breeding became profitable. The profitability of these relatively low HWRs suggested more New Zealand farmers should consider breeding their hoggets to improve farm profitability.

A second set of modelled scenarios identified that relatively smaller increases in FWR can achieve the same increases in profitability compared with much larger improvements in HWR. This comparison suggested that a farmer currently achieving the average industry FWR should prioritise improving FWR over hogget breeding as a means of increasing farm profitability.

Thirdly, the highest FWR (150%) and HWR (104%) modelled in the previous sets of scenarios were modelled together to represent a flock achieving high levels of reproductive success in all ewes and this modelled flock had the highest overall COS.

Fourthly, COS was the same for a flock with current industry average FWR (135%) and HWR (60%) as COS for a flock with FWR = 142% not breeding hoggets. This indicates an FWR which farmers should achieve to increase profitability before starting to breed their hoggets. Further, it indicates a FWR which a farmer who does not wish to breed hoggets could achieve to have the same profit as an ‘average’ farmer who breeds both mature ewes and hoggets.

## Figures and Tables

**Table 1:** Prices of sheep sold in 2019/20. Cull ewes, prime (sold direct to slaughter) lambs, and store (for a different farmer to grow to slaughter) lambs. Lambs from hoggets were sold store.

Sheep	Sale Timing	Price (NZD/Head)	Price Data Source
Ewes aged 3 years and older	Early December	138.75	[18]
Ewes aged 2 years		156.77	
Prime lambs	Early February	133.13 ^1^	[36]
	Mid-February	125.09 ^1^	
Store lambs	Mid-February	98.11	
Lambs born to hoggets	Mid-December	78.49	[4,36]

^1^ Per head prices for sales of prime lambs were estimated using weekly schedule prices on a per kg of carcass weight basis combined with carcass weight.

## Data Availability

The data presented in this study are available within the article.
